# Meropenem pharmacokinetics during relapsing peritonitis due to ESBL‐producing Enterobacteriaciae in a liver transplant recipient

**DOI:** 10.1002/ccr3.2398

**Published:** 2019-09-30

**Authors:** Kensuke Shoji, Jumpei Saito, Yusuke Oho, Shotaro Matsumoto, Satoshi Aoki, Akinari Fukuda, Seisuke Sakamoto, Mureo Kasahara, Edmund Capparelli, Isao Miyairi

**Affiliations:** ^1^ Division of Infectious Diseases Department of Medical Subspecialties National Center for Child Health and Development Tokyo Japan; ^2^ Department of Pharmacy National Center for Child Health and Development Tokyo Japan; ^3^ Division of Critical Care Medicine National Center for Child Health and Development Tokyo Japan; ^4^ Organ Transplantation Center National Center for Child Health and Development Tokyo Japan; ^5^ Division of Host‐Microbe Systems and Therapeutics UC San Diego La Jolla CA USA; ^6^ Department of Microbiology, Immunology, and Biochemistry University of Tennessee Health Science Center Memphis TN USA

**Keywords:** liver transplantation, meropenem, pediatrics, pharmacokinetics, prolonged infusion

## Abstract

We report on an 8‐year‐old girl with Wilson disease who developed three episodes of peritonitis due to extended‐spectrum beta‐lactamase‐producing *Escherichia coli* after liver transplantation. Massive ascites were thought to account for low meropenem concentrations with standard dosing. Extending the infusion achieved higher troughs, greater time above minimum inhibitory concentration.

## Graphical abstract

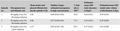

## 1 INTRODUCTION

Multidrug‐resistant (MDR) Enterobacteriaceae is a major clinical problem worldwide. It is known that the prognosis for infections due to MDR Enterobacteriaceae is worse than infections due to non‐MDR strains.^1^ Meropenem, classified as a carbapenem, is a first‐line antibiotic for the majority of MDR Enterobacteriaceae infections, including extended‐spectrum beta‐lactamase (ESBL)‐producing Enterobacteriaceae infections. Meropenem therapy has been associated with better outcomes compared to other broad‐spectrum antibiotics.^2^ Meropenem is a time‐dependent antibiotic, and its efficacy is dependent on the time above minimum inhibitory concentration (TMIC). In general, achieving a TMIC ≥ 40% is considered a sufficient pharmacodynamic (PD) target for meropenem. However, a recent study indicated that achieving a higher TMIC, such as 50%‐100% TMIC, was associated with favorable outcomes for severe infections in critically ill patients.^3^

There are several factors that might change the pharmacokinetics (PK) after solid organ transplantation (SOT), such as hemodynamic change due to surgery, blood loss, massive ascites, and inflammation. These changes might alter meropenem PK, resulting in different dosing requirements to maintain sufficient TMIC for severe bacterial infections due to MDR Enterobacteriaceae infection after SOT. However, there are no data regarding meropenem PK after liver transplantation (LT).

We present a case of bacterial peritonitis due to ESBL‐producing *Escherichia coli* in a pediatric LT recipient who had massive ascites. The patient experienced multiple relapses of peritonitis during the first four months post‐transplantation despite standard dosing and adequate duration of meropenem therapy.

## 2 CASE

An eight‐year‐old (29.9 kg) girl underwent deceased‐donor LT due to Wilson disease. She received ampicillin and cefotaxime as perioperative prophylactic antibiotics for 2 days. Immunosuppression after LT consisted of tacrolimus and methylprednisolone. After LT, massive ascites (up to 79 L over 7 days) developed, possibly due to small size graft and/or hepatic vein stenosis. On postoperative day (POD) 43, fever (maximum 40.0°C) and abdominal pain developed. The peripheral white blood cell count was elevated to 35,530/µL with 85% neutrophils. Total bilirubin, direct bilirubin, aspartate aminotransferase, alanine aminotransferase, creatinine, albumin, and C‐reactive protein levels were 4.18 mg/dL, 3.03 mg/dL, 50 U/L, 39 U/L, 0.34 mg/dL, 2.8 g/dL, and 5.45 mg/dL, respectively. Renal function was normal with an estimated glomerular function rate of 130 mL/min/1.73 m^2^. Ascites cell count was elevated at 1775/µL with 75% neutrophils and Gram staining revealed gram‐negative bacilli. Bacterial peritonitis was suspected, and piperacillin/tazobactam was initiated. Two days later (POD 45), exploratory laparotomy detected abdominal abscess and intensive peritoneal lavage was performed. MDR Enterobacteriaceae was isolated from the original ascites sample, and antibiotics were switched to meropenem (60 mg/kg/d) and gentamicin (5 mg/kg/d) every 24 hours. The isolate was identified as ESBL‐producing *E coli* (meropenem MIC ≤ 1.0 µg/mL), and gentamicin was discontinued. The dosing of meropenem was increased to 120 mg/kg/d every 8 hours (60‐minute infusion) on day 3 of treatment because of suspected underdosing due to massive ascites. The antibiotic dose was based on the patient's total weight. The patient's condition improved gradually, and meropenem was stopped after completion of 2 weeks of therapy (POD 59). However, fever, abdominal pain, and leukocytosis in blood (33 650/µL with 82% neutrophils) and ascites (3800/µL with 90% neutrophils) were observed 6 days after finishing the first course of meropenem‐based antibiotic therapy. Relapse of bacterial peritonitis was suspected, and meropenem (120 mg/kg/d every 8 hours, 60‐minute infusion) was reinitiated. The peritoneal drainage tube was replaced, and ascites culture was performed. The culture revealed ESBL‐producing *E coli* with the same antibiotic susceptibility pattern, confirming the diagnosis of relapsed peritonitis. The patient's condition improved after initiation of the second course of meropenem‐based antibiotic therapy. During this episode, normalization of ascites cell count was confirmed, and 3 weeks of meropenem therapy was completed. However, peritoneal abscess and peritonitis due to ESBL‐producing *E coli* relapsed again 12 days after completion of antibiotic therapy (POD 101). Meropenem was reinitiated at 120 mg/kg/d every 8 hours. This time, meropenem was administered using a prolonged (3 hours) infusion to achieve a longer time above MIC. Meropenem was continued for 4 weeks, but peritoneal effusion still persisted. Therefore, antibiotics were continued, but meropenem was switched to cefmetazole to reduce overall exposure to carbapenem antibiotics. Disappearance of abdominal abscess was confirmed by CT scan and abdominal ultrasound. Cefmetazole was stopped after 4 weeks of therapy and no relapse was observed thereafter. The amounts of pleural fluid and ascites were high with 4‐6 L/d throughout these three episodes.

### 2.1 Method for meropenem pharmacokinetic analysis

Remnant serum samples were scavenged for meropenem concentration determinations. These samples were analyzed using a validated liquid chromatography method with tandem mass spectrometric detection (LC/MS/MS).^4^ This LC/MS/MS method for determination of meropenem concentration was validated and met the requirements for specificity, sensitivity, linearity, recovery, precision, accuracy, and dilution integrity according to the European Medicines Agency guidelines. The PK data were fit to a two‐compartment model using the program WinBUGS version 1.4.3 (https://www.mrc-bsu.cam.ac.uk/software/bugs) to estimate the pharmacokinetic parameters in this patient. A previously validated and published pediatric population PK model of meropenem was provided as the PK parameter and variance priors for this analysis.^5^ This retrospective study was approved by the institutional review board at our hospital (NCCHD‐1328).

### 2.2 Result of meropenem pharmacokinetic analysis

**Figure 1:**
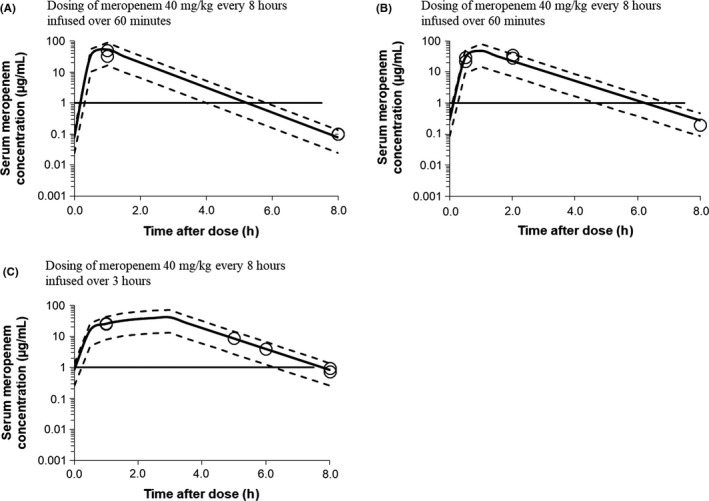
Estimated meropenem concentration‐time profile. A, first, B, second, C, third episode. Meropenem concentrations were estimated using the posterior distributions based on the presented Bayesian population approach. Dotted lines depict the upper and lower bounds of the 95% confidence interval of all simulations including the inferred measurement error

During each of the three peritonitis episodes, multiple serum meropenem concentrations were measured. The sampling timing and modeled meropenem concentration‐time profile are shown in Figure 1. Meropenem was administered as 40 mg/kg/dose every 8 hours, 60‐minute infusions in the first and second episodes and the same dose but 3‐hour infusions in the third episode. The PK and PD parameters in each episode are shown in Table 1. The modeled troughs were higher in the third episode with 3‐hour infusions than in the first and second episodes with 60‐minute infusions (0.82, 0.08, and 0.027 µg/mL, respectively). The estimated total meropenem clearance (CL) values were 0.44‐0.47 L/kg/h, and the estimated volumes of distribution at steady‐state (Vdss) were 0.43‐0.59 L/kg.

**Table 1:** Meropenem dose, infusion rate, and estimated pharmacokinetic and pharmacodynamic parameters for each episode

Episode no.	Meropenem dose and infusion rate	Mean ascites and pleural effusion during the episode (L/d)	Median (range) estimated meropenem trough concentration	% time above MIC^a^	Estimated mean (SD) total clearance (L/kg/h)	Estimated mean (SD) steady‐state volume of distribution (L/kg)
1	40 mg/kg every 8 h, 60‐minute infusion	4.84	0.08 (0.02‐0.20)	65.0	0.46 (0.01)	0.49 (0.03)
2	40 mg/kg every 8 h, 60‐minute infusion	6.08	0.27 (0.08‐0.46)	77.5	0.44 (0.01)	0.59 (0.08)
3	40 mg/kg every 8 h, 3‐hour infusion	4.13	0.82 (0.28‐1.38)	92.5	0.47 (0.01)	0.43 (0.06)

Abbreviations: MIC, minimum inhibitory concentration; SD, standard deviation.

a MIC was assumed to be 1 µg/mL.

The calculated TMIC (for meropenem MIC = 1 µg/mL) was highest (92.5%) in the third episode with prolonged infusion compared to 65.0% and 77.5% in the first and second episodes with 60‐minute infusions.

## 3 DISCUSSION

We encountered a case of relapsing peritonitis due to ESBL‐producing *E coli* in a pediatric LT recipient with massive ascites despite standard therapy that achieved a TMIC greater than 50%. Prolonged infusion of high‐dose meropenem was necessary to achieve a TMIC near 95%.

Severe infection due to MDR Enterobacteriaceae has been reported in immunocompromised patients including solid organ recipients, which leads to poor prognosis.^6^ Prompt initiation of appropriate antibiotics is essential in this situation. Meropenem has been shown to be clinically superior compared with other antibiotics 2; therefore, it has been used as a first‐line antibiotic for ESBL infections. Additionally, identification of the infectious focus is essential for treatment of bacterial infection. In general, removal of the focus by surgical debridement or drainage is recommended in patients with apparent source of infection such as an abscess. The guideline for treatment of secondary peritonitis recommends prompt initiation of antibiotics and surgical intervention when possible.^7^ From this perspective, this case received standard therapy for secondary peritonitis. However, the peritonitis relapsed in spite of standard therapy. Therefore, we suspected underexposure of meropenem due to alteration of the PK profile in the post‐transplant condition.

Several PK changes might occur after LT. LT recipients often experience large amounts of ascites or pleural fluid due to graft rejection, low albumin level, and alternation of blood flow. These changes lead to larger volumes of distribution for water‐soluble antibiotics. Body fluids, such as ascites, contain water‐soluble drugs that are often wasted through the drainage tube, leading to larger CL. In addition, inflammation due to transplantation or infection might lead to higher CL due to augmented renal clearance.^8^ Since the majority of beta‐lactam antibiotics including meropenem are highly water‐soluble, these medications are highly affected by PK changes due to transplantation. According to the PK analysis, the estimated total meropenem CL and the Vdss were approximately up to 1.4‐fold and 1.7‐fold higher, respectively, than in a reported population PK study in Japanese pediatric patients.^9^ Therefore, we considered that massive ascites and the patient's various symptoms might affect meropenem PK parameters.

Prolonged infusion was performed in this case, and higher TMIC was achieved. It is well known that prolonged or continuous infusion of antibiotics is effective to achieve higher TMIC. The clinical efficacy of prolonged or continuous infusion of antibiotics is still debatable. However, a recent meta‐analysis showed prognostic improvement in severe patients receiving prolonged infusions.^10^ The optimal TMIC in immunocompromised patients is still not well‐defined, but recent data supports that the higher TMICs, including up to 100%, may be associated with better clinical outcomes.^3^ In our case, while causality is still unclear, relapsing ESBL peritonitis was successfully treated by prolonged infusion with higher TMIC. Further investigations with a large number of patients are needed to clarify the clinical impact of prolonged infusion of antibiotics in pediatric immunocompromised patients with severe infection due to multidrug‐resistant organisms.

## CONFLICT OF INTEREST

All authors do not have any potential, perceived, or real conflict of interest.

## AUTHOR CONTRIBUTIONS

KS contributed to conceptualizing and designing the study and drafted the manuscript. JS and YO also contributed to conceptualizing and designing the study, data collection, PK analysis and revising the manuscript. SM, SA, AF, SS, and MK contributed to data collection and revising the manuscript. EC and IM contributed to the conceptualization and design of the study, revised the manuscript, and supervised the study. All authors approved the final manuscript as submitted and agree to be accountable for all aspects of the work.
